# Relationship between intensity of night shift work and antioxidant status in blood of nurses

**DOI:** 10.1007/s00420-012-0828-7

**Published:** 2012-11-23

**Authors:** Jolanta Gromadzińska, Beata Peplonska, Wojciech Sobala, Edyta Reszka, Wojciech Wasowicz, Agnieszka Bukowska, Jenny-Anne Lie

**Affiliations:** 1 Nofer Institute of Occupational Medicine, Lodz, Poland; 2 Institute of Occupational Health, Oslo, Norway

**Keywords:** Antioxidants, TBARS, Night shift work, Selenium, Light-at-night exposure

## Abstract

**Purpose:**

Light-at-night exposure can disrupt the human circadian rhythm via clock gene expressions. The circadian rhythm influences antioxidant enzymes’ activity and cellular mRNA levels of these enzymes. The employees working based on a shift system adjust to the changes occurring both on the cell level and on the level of the whole organism. Therefore, a question should be answered whether shift work disturbs oxidant–antioxidant balance and/or generates oxidative stress.

**Methods:**

A cross-sectional study was conducted among nurses selected from the Local Registry of the Chamber of Nurses and Midwives in Lodz: 359 nurses worked daily only and 349 working rotating night shifts. These two groups differed significantly in respect of age (*p* < 0.0001), menopausal status (*p* < 0.0001), and current smoking habit (*p* = 0.02). The average total work duration was significantly shorter (12.4 years) in nurses working currently rotating night shifts who worked significantly longer on night shifts than day-workers (26.6 years).

**Results:**

We found statistically significant higher red blood cell glutathione peroxidase in nurses working on night shifts (21.0 ± 4.6 vs. 20.0 ± 5.0 U/g Hb, *p* < 0.009) after adjusting for age, oral contraceptive hormone use, smoking, and drinking alcohol during last 24 h. Statistically significant lower vitamin A and E levels were found in the premenopausal women working in rotating system (0.690 ± 0.238 vs. 0.786 ± 0.262 μg/ml, *p* < 0.0001 for vitamin A and 10.93 ± 4.15 vs. 12.78 ± 4.75 μg/ml, *p* < 0.0001 for vitamin E). The marker of lipid peroxidation (TBARS concentration) was significantly lower in the premenopausal nurses than postmenopausal ones working day shifts only (2.06 ± 0.76 vs. 2.21 ± 0.80 nmol/ml, *p* < 0.038). We observed that erythrocyte GSH-Px activity rose statistically significant in nurses working more night shifts per month (*p* < 0.01).

**Conclusions:**

The results quoted above seem to support the existence of an association between light-at-night exposure and blood glutathione peroxidase activity in female shift workers. Nevertheless, in order to explain the mechanisms of this association, we need more studies.

## Introduction

A number of studies have investigated a possible role of environmental factors in cancer etiology. One of the factors of particular interest is exposure to light-at-night during the working hours of shift workers, which may cause sleep disruption and altered normal endocrine functions as well as health problems. According to Costa et al. (2010), the data collected in 2005 by the European Foundation for the Improvement of Living and Working Conditions showed that 21.9 % of men and 10.7 % of women work within a shift system that includes evening and night work. Seven per cent of shift workers permanently work at night (European Foundation for the Improvement of Living and Working Conditions 2007). It has been shown that shift work together with the abnormal light–dark cycle connected with it cause adverse health effects. Short-term disturbances in the normal sleep–wake cycle give reversible symptoms called a “jet-lag” syndrome (trouble with sleeping, fatigue, lack of appetite). Long-term altered light–night cycle causes chronic sleep deprivation, gastrointestinal and cardiovascular disorders, and adverse pregnancy outcome (Knutsson 2003). In several recent studies, an increase has been shown in the risk of developing cancer, in particular breast, endometrial and colon cancer, among shift workers (Schernhammer and Schulmeister 2004; Hansen 2006).

A review of epidemiological studies devoted to cancer risk in shift workers performed by Kolstad (2008) and Pauley (2004) demonstrated 36–60 % higher rates of breast cancer risk among this population. In 2007, the International Agency for Research on Cancer classified night shift work as a probable carcinogen (group 2A), based on limited evidence from human studies and adequate evidence from animal experiments (Straif et al. 2007).

Light exposure during night hours changes melatonin secretion and can disrupt the human circadian rhythm via melatonin secretion (Mirick and Davis 2008). A circadian rhythm disruption induces altered endocrine functions—possible changes in the regulation of reproductive hormone receptors—and thus it is an important factor in the etiology of hormone-related diseases, for example, breast or prostate cancer (Mirick and Davis 2008; Grant et al. 2009). Alterations in the circadian system are observed in tumor tissues during experiments with tumor bearing animals as well as in cancer patients (Mormont and Levi 1997; Cardona 2004).

The imbalance in oxidant–antioxidant levels is known to be a possible key factor in the pathogenesis of many human diseases, including breast cancer. To protect cells from oxidative damage, organisms have generated several defense mechanisms, namely enzymatic and non-enzymatic ones to remove reactive oxygen species from extra- and intracellular spaces (Yeh et al. 2005; Yeon et al. 2011).

In many animal experiments, it has been shown that expression and/or activity of oxidative and antioxidative enzymes depend on the circadian rhythm (Kolanjiappan and Manoharan 2005; Baydas et al. 2002; Jimenez-Ortega et al. 2009). The circadian rhythm influences antioxidant enzymes’ activity and cellular mRNA levels of these enzymes: glutathione peroxidase, superoxide dismutase (cellular and mitochondrial fraction), catalase, nitric oxide synthase, and heme oxidase (Mayo et al. 2002; Rodriguez et al. 2004; Jimenez-Ortega et al. 2009). The mechanism is unknown, but it probably follows the activation of transcriptional factors in the promoter region of antioxidative enzyme genes (Rodriguez et al. 2004).

Exposure to light-at-night results in altered endocrine functions (Mirick and Davis 2008). This is followed by generation of oxidative stress and many health disorders originating from shift work. This is followed by generation of oxidative stress and many health disorders, whose source originally is shift work. The employees working in a shift system adjust to the changes occurring both on the cell level and on the level of the whole organism. However, it has not yet been investigated whether night shift work induces changes in the concentrations/activities of antioxidants as factors with the proven association with cancer development.

The present study was carried out in a population of nurses and midwives working currently under different work schedules in order to investigate the relationship between the blood antioxidant levels (glutathione peroxidase and superoxide dismutase activity, plasma selenium, vitamin A and E levels), thiobarbituric acid reactive substances (TBARS) as a marker of pro-oxidative processes and lifestyle habits as well as work-related factors: current rotating night shift work status and frequency as well as total night shift history, age, and menopausal status.

## Materials and methods

The cross-sectional study was conducted among nurses (aged 40–60) selected from the Local Registry of the Chamber of Nurses and Midwives in Lodz. Healthy women without any chronic diseases were selected for this study. After obtaining a written informed consent from each participant, information was collected during an in-person interview, regarding their occupational history, demographic characteristics, medical and reproductive history, physical activity, smoking habits, and sleep quality.

Based on the data gathered via the interview, the average frequency of night shifts at the current job was analyzed in the following categories: 0, 2, 4 and 8, or more nights per month. Additionally, we calculated the intensity of the work performed on night shifts during the whole work period.

Blood samples were collected between 06:00 and 10:00 a.m from each participant into S-Monovette^®^ test tubes with lithium heparin as anticoagulant. In the case of night shift workers, blood collection was synchronized with the night shift, and the blood samples were collected at the end of night shift.

Glutathione peroxidase activity (GSH-Px) was determined by the method of Paglia and Valentine (1967) with *t*-butyl hydroperoxide as substrate. Superoxide dismutase (SOD) was assayed with the use of the method based on the inhibition of reduction of nitroblue tetrazolium in the presence of xanthine and xanthine oxidase (Beauchamp and Fridovich 1971). Lipid peroxidation was estimated from the measurements of TBARS levels in plasma using the method modified by Wasowicz et al. (1993).

The plasma selenium concentration was determined by graphite furnace atomic absorption spectrometry (GFAAS) (Neve 1991). The method was validated using the reference material (lyophilized human reference serum samples of Seronorm from Nycomed Pharma AS, Oslo, Norway) and through participation in the interlaboratory comparison trials.

Vitamin E and A levels were determined by the HPLC system integrated with UV–VIS detector range 190–800 nm (Grzelinska et al. 2007).

### Statistical analysis

The data from the biochemical analyses was expressed as a mean and a standard deviation.

Characteristics of the study groups were compared using the Pearson’s chi-squared test and the Student’s *t* test. Linear regression model was used to analyze the relationship between antioxidants and markers of oxidative stress and night shift work. Multivariate linear regression was applied to adjust for age, oral contraceptive hormone use, smoking, and drinking alcohol during last 24 h as potential confounders. Following that, the interaction between night shift work and menopausal status was investigated. We used robust linear regression to validate our results against the outliers.

STATA 11 software was used to conduct all statistical analyses.

## Results

The characteristics of the studied population comprising nurses and midwives are listed in Table 1. In the investigated group, at the time of the research, 359 nurses worked only daytime and 349 worked currently on rotating night shifts. These two groups differed significantly as for age (*p* < 0.0001), menopausal status (*p* < 0.0001), and current smoking habits (*p* = 0.02). The average total work duration was significantly shorter (27.5 ± 6.6 years) in nurses working currently on rotating night shifts than in day-workers (29.2 ± 6.3 years) (data not shown). The current night shift workers had, however, worked night shifts significantly longer (26.6 vs. 12.4 years).

**Table 1:** Selected characteristics of the nurses and midwives in the cross-sectional study

	Day-workers *n* = 359	Rotating shifts workers *n* = 349	*p*
Age, years	50.2 ± 5.3 (40.1–61.1)	48.3 ± 5.2 (39.5–60.2)	<0.0001
BMI, kg/m^2^	27.1 ± 4.7 (18.5–48.3)	27.1 ± 4.6 (16.4–45.2)	0.98
Total night shift work, years	12.4 ± 8.3 (0–37.3)	26.6 ± 7.3 (4.6–42.3)	<0.0001
*Total night shift work (categories)*			
<5 years	76 (21.2)	0	0.0001
6–15 years	147 (40.9)	30 (8.6)	
>15 years	136 (37.9)	319 (91.4)	
*Current night shift work frequency per month*			
<2 nights		2 (0.58 %)	
2–4 nights		19 (5.44 %)	
5–8 nights		320 (91.69 %)	
>8 nights		8 (2.29 %)	
*Smoking*			
Never smokers	146 (41.8 %)	155 (43.0 %)	0.02
Past smokers	81 (23.2 %)	110 (30.6 %)	
Current smokers	122 (35.0 %)	95 (26.4 %)	
*Menopausal status*			
Pre-	185 (51.5 %)	225 (65.7 %)	<0.0001
Post-	174 (48.5 %)	124 (34.3 %)	
*Current oral contraceptives or sex hormone use*			
Yes	89 (24.8 %)	80 (23.0 %)	0.513
No	270 (75.2 %)	269 (77.0 %)	

The average period of employment under shift work conditions of women currently working rotating night shifts was significantly longer (24.20 ± 7.03 years) than in nurses working currently day shifts (11.98 ± 8.08 years). Almost all the nurses and midwives who were current day-workers had worked previously rotating night shifts. However, all women in that group did not work rotating shifts during the last 5 years. In the day-worker group, only 10 of the women did not work rotating shifts. The majority (91.4 %) of currently working rotating night shift women were exposed more than 15 years to light-at-night, while about 38.0 % of women currently working day shifts, worked more than 15 years under light-at-night exposure. Among the nurses currently working rotating shifts, nearly 92 % work 5–8 night shifts per month, 21 women work up to 4 night shifts per month, and 8 women work above 8 night shifts per month (Table 1).

Table 2 shows markers of oxidative stress in nurses and midwives according to work system. We found statistically significant higher red blood cell glutathione peroxidase activity (RBC GSH-Px) in nurses working night shifts (21.0 ± 4.6 vs. 20.0 ± 5.0 U/g Hb, *p* < 0.009), after adjustment for age, oral contraceptive hormone use, smoking, and drinking alcohol during last 24 h.

**Table 2:** Antioxidant and TBARS levels in the blood of nurses and midwives working currently within the rotating night shifts system or during the day only

Parameters	Day shift *n* = 359 (185/174)	Rotating nights *n* = 349 (225/124)	*p* crude	*p* adjustment*
*Plasma GSH*-*Px activity*, U/ml				
All	0.188 ± 0.030	0.188 ± 0.033	0.952	0.974
Premenopause	0.182 ± 0.032	0.189 ± 0.030	0.029	0.137
Postmenopause	0.193 ± 0.032	0.185 ± 0.030	0.024	**0.037**
*p* (pre: postmenopause)*	**0.001**	0.310		
*RBC GSH*-*Px activity*, U/g Hb				
All	20.0 ± 5.0	21.0 ± 4.6	0.006	**0.009**
Premenopause	19.4 ± 4.7	21.0 ± 4.8	0.001	**0.011**
Postmenopause	20.6 ± 5.1	21.0 ± 4.4	0.554	0.331
*p* (pre: postmenopause)*	**0.011**	0.950		
*RBC SOD activity*, U/mg Hb				
All	6.96 ± 1.40	6.89 ± 1.54	0.526	0.741
Premenopause	6.88 ± 1.46	6.86 ± 1.57	0.909	0.562
Postmenopause	7.04 ± 1.33	6.97 ± 1.49	0.539	0.768
*p* (pre: postmenopause)*	0.259	0.640		
*Plasma selenium*, μg/l				
All	56.7 ± 11.4	55.0 ± 11.4	0.044	0.435
Premenopause	56.2 ± 11.5	54.1 ± 10.8	0.044	0.650
Postmenopause	57.3 ± 11.2	56.7 ± 13.1	0.687	0.444
*p* (pre: postmenopause)*	0.404	0.053		
*Plasma vitamin E*, μg/ml				
All	11.42 ± 4.72	11.53 ± 4.41	0.761	0.099
Premenopause	10.96 ± 4.97	10.93 ± 4.15	0.937	0.099
Postmenopause	12.00 ± 5.18	12.78 ± 4.75	0.219	0.099
*p* (pre: postmenopause)*	**0.023**	**0.0001**		
*Plasma vitamin A*, μg/ml				
All	0.700 ± 0.248	0.722 ± 0.231	0.234	0.170
Premenopause	0.690 ± 0.260	0.690 ± 0.238	0.957	0.671
Postmenopause	0.711 ± 0.160	0.786 ± 0.262	0.005	**0.003**
*p* (pre: postmenopause)*	0.452	**0.0001**		
*Plasma TBARS*, nmol/ml				
All	2.14 ± 0.79	2.11 ± 0.78	0.648	0.767
Premenopause	2.06 ± 0.76	2.21 ± 0.80	0.991	0.624
Postmenopause	2.21 ± 0.80	2.22 ± 0.82	0.957	0.908
*p* (pre: postmenopause)*	**0.038**	0.057		

Results expressed as mean ± SD

Statistically significant differences are given in bold

* Adjusted for age, oral contraceptive hormone use, smoking, and drinking alcohol during the last 24 h

When antioxidant parameters in blood were analyzed according to menopausal status, we found statistically lower plasma GSH-Px activity and RBC GSH-Px activity in premenopausal nurses as compared with postmenopausal ones (19.4 ± 4.7 vs. 20.6 ± 5.1 U/g Hb, *p* < 0.011). Besides, statistically significant lower vitamin A and E levels were found in the premenopausal women working in the rotating shift system (0.690 ± 0.238 vs. 0.786 ± 0.262 μg/ml, *p* < 0.0001 for vitamin A and 10.93 ± 4.15 vs. 12.78 ± 4.75 μg/ml, *p* < 0.0001 for vitamin E). The marker of lipid peroxidation, TBARS concentration, was significantly lower in the premenopausal nurses than in postmenopausal ones working day shifts only (2.06 ± 0.76 vs. 2.21 ± 0.80 nmol/ml, *p* < 0.038).

When the premenopausal nurses were categorized into day shift only and working on rotating night shift, we found statistically higher values for erythrocyte glutathione peroxidase activity in the rotating night shift nurses (Table 2). Erythrocyte GSH-Px activity was 21.0 ± 4.8 U/g Hb in premenopausal rotating night shift nurses, compared with 19.4 ± 4.7 U/g Hb in day shift workers (*p* < 0.011). As for plasma GSH-Px activity, the values for menopausal nurses working in rotating system were 0.185 ± 0.030 U/ml and for working day shift only was 0.193 ± 0.032 U/ml, *p* < 0.037.

The postmenopausal nurses working in a rotating system had higher plasma vitamin A levels compared with nurses working day shifts only (Table 2). Erythrocyte glutathione peroxidase activity was higher in premenopausal nurses working rotating night shifts than in the premenopausal subjects working days only.

Based on the data collected via the interview, we calculated the total number of night shifts during the subjects’ working period. We did not find any relationship between the measured parameter of oxidative stress and cumulative shift work during the whole occupational history (Table 3).

**Table 3:** Antioxidants and TBARS levels in the blood in relation to the cumulative number of night shift work in nurses currently working in rotating system (*n* = 349)

Parameters	Total rotating shifts number during the whole work life	
	<300 months *n* = 147	>300 months *n* = 202
Plasma GSH-Px activity, U/ml	0.188 ± 0.030	0.188 ± 0.035
		0.954
		0.936*
RBC GSH-Px activity, U/g Hb	20.8 ± 5.0	21.2 ± 4.3
		0.877
		0.856*
RBC SOD activity, U/mg Hb	7.01 ± 1.60	6.81 ± 1.49
		0.8928
		0.837*
Plasma selenium, μg/l	54.1 ± 10.7	55.7 ± 11.8
		0.516
		0.745*
Plasma vitamin E, μg/ml	10.47 ± 4.25	12.35 ± 4.42
		0.314
		0.179*
Plasma vitamin A, μg/ml	0.666 ± 0.247	0.763 ± 0.209
		0.398
		0.542*
Plasma TBARS, nmol/ml	2.04 ± 0.71	2.16 ± 0.82
		0.736
		0.669*

Results expressed as mean ± SD

* After adjustment for age, oral contraceptive hormones use, current HRT use, smoking habits, and drinking alcohol during the last 24 h

**Fig. 1:**
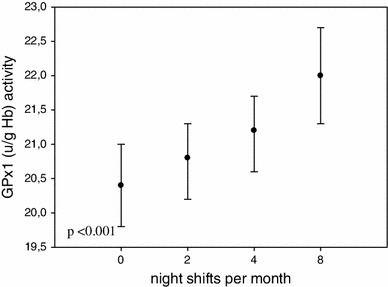
Association between night shift work frequency and RBC GSH-Px activity. Comparison of RBC GSH-Px activity among nurses 0—working on day shift only (*n* = 359), 2—working less than 2 nights/month (*n* = 2), 4—working 2–4 night shifts/months (*n* = 19), 8—working 5–8 night shifts/month (*n* = 320). Statistical analysis after adjustment for age, oral contraceptive hormone use, smoking, and drinking alcohol during the last 24 h

**Fig. 2:**
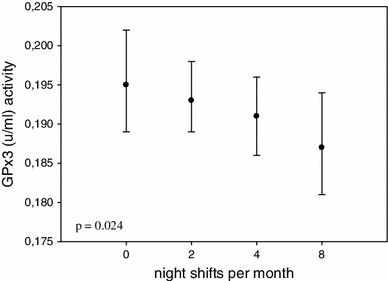
Association between night shift work frequency and plasma GSH-Px activity in the postmenopausal women. Comparison of plasma GSH-Px activity among postmenopausal nurses 0—working on day shift only (*n* = 174), 2—working less than 2 nights/month (*n* = 2), 4—working 2–4 night shifts/months (*n* = 12), 8—working 5–8 night shifts/month (*n* = 102). Statistical analysis after adjustment for age, oral contraceptive hormone use in the past, smoking, and drinking alcohol during the last 24 h

The association between night shift work frequency per month and the antioxidant enzymes activity is presented in Fig. 1. We observed that the erythrocyte GSH-Px activity rose statistically significant in nurses working on more night shifts per month (*p* < 0.001). The association between plasma GSH-Px activity and night shift work differed significantly between pre- and postmenopausal nurses: it was higher (*p* < 0.008) in the premenopausal subjects and lower (*p* < 0.024) in the postmenopausal ones (Fig. 2).

## Discussion

A number of clinical and experimental studies have indicated that exposure to a number of physical and/or chemical agents may generate reactive oxygen species (ROS) and promote oxidative stress. ROS react with unsaturated fatty acids of cell membranes, as well as with proteins and nucleic acid and may play an indirect role in disease development (Valko et al. 2004). Mammalian cells have different antioxidant systems including various antioxidative enzymes comprise the necessary trace elements (Se, Zn, Cu, Mn), as well as low-molecular-weight antioxidants: vitamin A, E, C, glutathione, uric acid, etc. (Polat et al. 2002). It has been observed in animal experiments that antioxidant enzyme activities and their gene expression exhibit cyclic 24 h rhythm under normal light–dark conditions. Experiments with rats and chicken have shown that brain GSH-Px and SOD activity is higher at night-time than at day-time (Pablos et al. 1998; Albarrán et al. 2001).

On the other hand, Baydas et al. (2001, 2002) found that constant exposure to light decreases the GSH-Px activity in rat brain, liver, and kidney. Circadian variations of brain enzymes have been described for many redox state controlling enzymes (Jimenez-Ortega et al. 2009). Twenty-four hour changes in the enzyme activity suggest that this cycle may be dependent on the circadian melatonin rhythm (Baydas et al. 2002).

In the group of 349 nurses working within a rotating night and day shifts system, we found significantly higher RBC GSH-Px activity (*p* < 0.009 after adjustment for age, oral contraceptive hormone use, smoking and drinking alcohol during the last 24 h). Moreover, a progressive increase was found to occur in the RBC GH-Px activity related to the frequency of night shifts per month (Fig. 1, *p* < 0.001). Such clear, statistically significant, changes were demonstrated only for the activity of RBC GSH-Px in the premenopausal nurses. For the postmenopausal subjects, the changes were not statistically significant.

The remaining studied parameters (markers of antioxidative processes and TBARS) did not differ between study groups working in different work systems. In female workers, estrogen level is an additional factor affecting the redox potential. Women before menopause are protected from the toxic effects of reactive oxygen species, because estrogens play an important role as endogenous antioxidants (Krstevska et al. 2001). It has been postulated, although a final proof is still missing, that estrogens may have protective effects against lipid peroxidation (Brown et al. 2000; Chiang et al. 2004). Studies performed on rats or women receiving HRT demonstrated a quite opposite effect: increase in blood lipid peroxides and/or decrease in plasma B-carotene—precursor of vitamin A (Berg et al. 1997). Ha and Smith (2009) found significantly higher GSH-Px activity in plasma and RBC of healthy postmenopausal women aged 70.9 ± 3.5 years, compared with the premenopausal ones. The Se level in their study did not differ between the pre- and postmenopausal women. Considering that the accessible results are divergent, and that there are few studies on the effects of shift work in healthy volunteers, we have decided to analyze our results with reference to the menopausal status of our subjects. Higher erythrocyte and plasma GSH-Px activities and elevated vitamin E levels have been found in the postmenopausal nurses working currently day shift as compared with the premenopausal ones. The changes in those antioxidants are accompanied by increased TBARS levels in the blood plasma of the postmenopausal women.

Our comparison of GSH-Px activity both in plasma and erythrocytes of women working in the shift work system versus women working day shifts only showed reduced plasma GSH-Px in postmenopausal nurses. The RBC GSH-Px activity in premenopausal nurses working rotating shifts was significantly higher than in those working only day shifts. Plasma GSH-Px and RBC GSH-Px are quite different proteins coded on different chromosomes and dominantly synthesized by different tissues.

GSH-Px protein is synthesized mainly in the kidneys, but also in the liver and other organs and released to the blood. Therefore, we may assume that the diurnal cycle of these organs affects the final activity of plasma GSH-Px. Unfortunately, such results for humans are not accessible; therefore, it is difficult to guess how light-at-night exposure may affect renal circadian cycle to modify plasma GSH-Px activity. As the changes in plasma GSH-Px activity were analyzed immediately after termination of the exposure and differences were detected only in the postmenopausal nurses, it seems reasonable to assume that the lower activity of plasma GSH-Px results from oxidative stress associated with lower estrogen concentrations and with the light-at-night exposure of that group of women. Such assumption is supported also by gradual decrease in plasma GSH-Px activity in relation to frequency of night shift work per month (Fig. 2).

It is also speculated that, due to the increased oxidative stress during menopause, estrogens can act as a specific modulator of the GSH-Px activity (Ha and Smith 2009). There is evidence that the GSH-Px activity may be directly inactivated by ROS, and, at the same time, ROS may activate the transcription of mRNA GSH-Px and the synthesis of new GSH-Px molecules (Miyamoto et al. 2003). Thus, at low concentrations of melatonin, as a result of light-at-night exposure, another pathway of this protein synthesis may be activated.

The influence of light-at-night exposure and melatonin level changes on erythrocytic GSH-Px activity is more complicated. Human mature erythrocytes do not include cell nuclei, do not have mRNA GSH-Px and do not synthesize the GSH-Px protein. The observed changes in the enzyme activity are results of the influence of circadian rhythm dysregulation on immature erythrocytes. RBC GSH-Px activity detected in the present study represents the resultant of the exposure of the study nurses during the last 120 days. As the increase in RBC GSH-Px activity has been recorded in the whole study group of nurses working in a rotating shift system and, in addition, it is directly proportional to the frequency of night shift work per month, it is reasonable to suppose that some other mechanisms are involved.

In some epidemiological studies, an association between night shift work related to circadian rhythm dysregulation and increased risk of developing cancer, in particular breast cancer, has been observed (Schernhammer et al. 2001). Disruption of the circadian rhythm has been associated with cancer development in animal experiments (Levi and Schibler 2007). Melatonin plays an important role in the regulation of the circadian rhythm and has been found to make an effective antioxidant and scavenger of ROS (Reiter et al. 1995). Light-at-night exposure suppresses the melatonin synthesis, decreases the GH-Px activity, and probably also that of other enzymes from the antioxidative pathway. It also influences cellular oxidative equilibrium (Rodriguez et al. 2004). Decreased antioxidative potential facilitates generation of stress.

Davis et al. (2001) suggested that lowered nocturnal melatonin level in subjects exposed to light-at-night could increase the release of estrogens from ovaries and thus it could stimulate the turnover of epithelial stem cells, one of the factors responsible for breast cancer development. The results obtained in this study should make the basis to conduct an extensive research on the relation of the concentrations/activity of antioxidants with shift work. It is especially so in light of the data showing that high concentration of plasma Se is a protective factor in estimating the risk of cancer development, and high RBC GSH-Px activity is related to increased risk of breast cancer development (Rajneesh et al. 2008; Moradi et al. 2009).

Although interesting, at the present stage of the research, we have difficulties to explain the statistically significant higher levels of vitamin A and E in the plasma of postmenopausal women, irrespective of the work system. It may be explained by a mechanism meant to compensate for reduced antioxidant potential due to low estrogen levels. At the same time, the differences in the vitamin concentration between young females and postmenopausal ones may be linked to dietary habits—a reduced intake of food, limited consumption of certain products, food interactions with drugs, etc. So far, data are too limited to suggest any relationship between levels of vitamins A and E and shift work system. The results from the present study support an association between exposure to light-at-night and altered levels of some antioxidant levels in female shift workers.
